# A dataset for sustainability assessment of agroecological practices in a crop-livestock farming system

**DOI:** 10.1016/j.dib.2021.107078

**Published:** 2021-04-22

**Authors:** Julia Jouan, Matthieu Carof, Rim Baccar, Nathalie Bareille, Suzanne Bastian, Delphine Brogna, Giovanni Burgio, Sébastien Couvreur, Michał Cupiał, Marc Dufrêne, Benjamin Dumont, Philippe Gontier, Anne-Lise Jacquot, Jarosław Kański, Serena Magagnoli, Joanna Makulska, Guénola Pérès, Aude Ridier, Thibault Salou, Fabio Sgolastra, Anna Szeląg-Sikora, Sylwester Tabor, Barbara Tombarkiewicz, Andrzej Węglarz, Olivier Godinot

**Affiliations:** a SAS, INRAE, Institut Agro, 35042 Rennes, France; b USC 1432 LEVA, Ecole Supérieure d'Agricultures, INRAE, SFR 4207 QUASAV, 49100 Angers, France; c INRAE, Oniris, BIOEPAR, 44300 Nantes, France; d ULiège Gembloux Agro-Bio Tech, TERRA Research and Teaching Center, B-5030 Gembloux, Belgium; e DISTAL, Alma Mater Studiorum Università di Bologna, 40126 Bologna, Italy; f USC 1481 URSE, Ecole Supérieure d'Agricultures, INRAE, 49007 Angers, France; g University of Agriculture in Krakow, Faculty of Production and Power Engineering, 30-149 Kraków, Poland; h PEGASE, INRAE, Institut Agro, 35042 Rennes, France; i Faculty of Animal Science, University of Agriculture in Krakow, 30-0599 Kraków, Poland; j SMART-LERECO, INRAE, Institut Agro, 35042 Rennes, France; k ITAP, Univ Montpellier, INRAE, Institut Agro, 34060 Montpellier, France

**Keywords:** Sustainability indicators, Crop-livestock integration, Systems approach, Transition management

## Abstract

This article presents data designed by European researchers who performed a literature review and interpreted the results to determine impact factors of many agroecological practices on a wide variety of sustainability indicators. The impact factors are represented in a matrix that connects practices to indicators. The indicators are related to environmental, economic and social sustainability of a typical European integrated crop-livestock farm. The data are included in the serious game SEGAE to learn agroecology, as described in “SEGAE: a serious game to learn agroecology” [1]. The data can be modified to adapt the game to other agricultural systems. Finally, the data can be re-used in research projects as a basis to assess impacts of agroecological practices.

## Specifications Table

**Table:** 

Subject	Agricultural and Biological Sciences (General)
Specific subject area	Assessment of impacts of agroecological practices on environmental, economic and social sustainability of an integrated crop-livestock farm
Type of data	Table
How data were acquired	Literature review Expert assessment
Data format	Raw and analyzed
Parameters for data collection	No specific condition
Description of data collection	Data were obtained from a literature review performed by researchers from six European universities. These experts interpreted results of the literature review to determine impact factors of many practices on a set of indicators related to the three pillars of sustainability.
Data source location	Institution: Institut Agro City/Town/Region: Rennes Country: France Most data were obtained from a literature review that focused on European agricultural systems but also included studies from other temperate regions. All sources of primary data are available in the dataset.
Data accessibility	Repository name: Mendeley Data Data identification number: fp3dvvm3×6 Direct URL to data: http://dx.doi.org/10.17632/fp3dvvm3x6.2
Related research article	J. Jouan, M. Carof, R. Baccar, N. Bareille, S. Bastian, D. Brogna, G. Burgio, S. Couvreur, M. Cupiał, M. Dufrêne, B. Dumont, P. Gontier, A.-L. Jacquot, J. Kański, S. Magagnoli, J. Makulska, G. Pérès, A. Ridier, T. Salou, F. Sgolastra, A. Szeląg-Sikora, S. Tabor, B. Tombarkiewicz, A. Węglarz, O. Godinot, SEGAE: a serious game to learn agroecology, Agricultural Systems. In Press

## Value of the Data

• The data highlight impacts of various agroecological practices on a set of indicators related to environmental, economic and social sustainability of an integrated crop-livestock farm.
• The data are included in the serious game SEGAE to learn agroecology. Students or professionals in the agricultural sector can use the game autonomously or while supervised by a teacher.
• The data can be modified to adapt the game to other agricultural systems. It can be used in research projects as a basis to describe impacts of agroecological practices. The set of indicators can also be used to perform integrated assessment of farming systems separately from the game.

## 1 Data Description

A serious game called SEGAE was created by six European universities in 2020 [1]. The game aims to improve the process of learning agroecology. Based on a modeling framework, it includes a graphical interface and a matrix that are connected by a calculation engine programmed in JavaScript. The matrix quantifies impacts of agroecological practices on many indicators in a synthetic way using an output-oriented approach [2]. Unlike a process-based approach, which mechanistically represents biological processes in a farming system, the output-oriented approach focuses directly on impacts of practices on indicators. The output-oriented approach can thus be likened to an empirical approach at the farm scale. The main advantage of this approach is to summarize in a matrix the impacts of practices on relevant indicators while keeping the model simple.

The database available with this article is an adapted version of the matrix in the game. It was (i) simplified to ease reading and (ii) supplemented with the references on which it is based. This database is available in a Microsoft® Excel workbook (SEGAEmatrix.xlsx), which contains seven worksheets:

- A brief User Manual to get started with the matrix
- Five intermediate worksheets contain the game's matrix and default values of parameters and indicators. The methods used to determine impact factors are specified, as are their references. The matrix was divided into four worksheets to separate indicators related to crop production, animal production, the environment and socio-economic aspects of the farming system.
- The final worksheet provides the details of the references cited in the intermediate worksheets.

## 2 Experimental Design, Materials and Methods

### 2.1 General approach to define the impact of practices on indicators

The categories of practices included in the matrix were chosen and adapted from two studies [3], [4] by an interdisciplinary group of researchers working in the SEGAE project. These researchers come from six universities: ESA (Ecole Supérieure d'Agricultures, Angers, France), Institut Agro (Rennes, France), Oniris (Nantes, France), ULiège Gembloux Agro-Bio Tech (Gembloux, Belgium), University of Agriculture in Krakow (Krakow, Poland) and Università di Bologna (Bologna, Italy). Their expertise covers agronomy and plant sciences, veterinary science, animal science (including social aspects), ecology and economics. The indicators were chosen to cover the three pillars of sustainability based on previous frameworks (e.g., Sadok et al. [5]), while remaining understandable by students and relevant to farmers.

The impact factors were determined in different ways:

1) found in original studies described in peer-reviewed articles
2) determined by analyzing several scientific articles or local technical documents
3) calculated using specific tools (e.g., scientific models or software)
4) estimated by our expert assessment in the associated fields
5) used only for model internal model calculations, with no influence on sustainability indicators

**Fig. 1:**
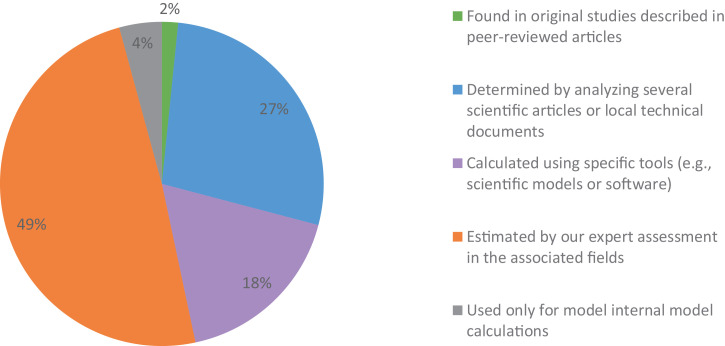
The distribution of approaches used to determine impact factors.

When possible, we favored ways 1 (2% of impact factors) and 2 (27%) and used way 4 (49%) only when we could not do otherwise (Fig. 1).

### 2.2 Examples of approaches used to determine impact factors

❖ ***Found in original studies published in peer-reviewed articles***

The impact factors derived from scientific studies were either found directly in the study (i.e., the value of the factor corresponded to a result of the study) or derived from a simple calculation. For example, the impact factor of “no tillage practices” on “crop yields” was defined by calculating the mean of the values “No-till as percentage of ploughed” available in Soane et al. [6] (the extreme value “200%” was excluded from this calculation).

❖ ***Determined by analyzing several scientific articles or local technical documents***

In other cases, impact factors were determined after analyzing several scientific studies or local technical documents. For example, the impact factors of agroecological practices related to soil cover, “Fall cover crop destroyed early spring” and “Permanent soil cover”, on the abundance of “natural enemies” was determined from two studies [7], [8]. The impact factors correspond to a range of, respectively, +5% and +10% of abundance of natural enemies compared to the default practice “Fall cover crop destroyed before winter”.

❖ ***Calculated using models***

Some impact factors were calculated using models or scientific methods. For example, feed requirements of cows were calculated by researchers from the University of Agriculture in Krakow using the software INRAtion [9]. Feed requirements of heifers, bulls and steers were calculated by researchers from ESA, using Forage Rummy© [10]. The nitrogen budget of cropping systems (nitrogen needs of crops and grasslands, nitrogen supply from organic matter mineralization, fertilizers, fixation and crop residues) was calculated using the COMIFER method [11]. Impacts of crop-related practices on annual working time were determined by researchers from Institut Agro. To do so, each crop was defined by a succession of standard technical operations, and alternative ones corresponding to the practice implemented. The standard operations were determined by a literature review [12], [13] and the alternative ones by expert assessment. The associated working times were then calculated using the Agribalyse database [14] and compiled in the matrix.

❖ ***Estimated by expert assessment***

Several impact factors were defined using expert assessment. For example, the routine work related to animal production was determined by researchers from Institut Agro. To do so, standard routine work for each group of tasks (e.g., milking, feeding cows) was identified based on a case study [15] and national and local data on routine work [16]. Then, the impacts of practices related to animal production on routine work were determined by the experts. In addition, the water quality index due to pesticides is a qualitative indicator that ranges from 0-5, based on practices known to increase or decrease risks of pesticide transfer to water. Practices were classified in five categories by expert assessment: much greater risk of transfer (−1 point), greater risk (−0.5 point), no effect (0 point), lower risk (+0.5 point) and much lower risk (+1 point).

## Declaration of Competing Interest

The authors declare that they have no known competing financial interests or personal relationships which have, or could be perceived to have, influenced the work reported in this article.

## Appendix Supplementary Materials

Image, application 1
